# Easy and Fast Reconstruction of a 3D Avatar with an RGB-D Sensor

**DOI:** 10.3390/s17051113

**Published:** 2017-05-12

**Authors:** Aihua Mao, Hong Zhang, Yuxin Liu, Yinglong Zheng, Guiqing Li, Guoqiang Han

**Affiliations:** School of Computer Science & Engineering, South China University of Technology, Guangzhou 510006, China; dragonaxz@yahoo.com (H.Z.); liuyuxin2934@gmail.com (Y.L.); ylong.zh@gmail.com (Y.Z.); ligq@scut.edu.cn (G.L.); csgqhan@scut.edu.cn (G.H.)

**Keywords:** human body, 3D reconstruction, RBG-D sensor, scanning strategy, alignment algorithm

## Abstract

This paper proposes a new easy and fast 3D avatar reconstruction method using an RGB-D sensor. Users can easily implement human body scanning and modeling just with a personal computer and a single RGB-D sensor such as a Microsoft Kinect within a small workspace in their home or office. To make the reconstruction of 3D avatars easy and fast, a new data capture strategy is proposed for efficient human body scanning, which captures only 18 frames from six views with a close scanning distance to fully cover the body; meanwhile, efficient alignment algorithms are presented to locally align the data frames in the single view and then globally align them in multi-views based on pairwise correspondence. In this method, we do not adopt shape priors or subdivision tools to synthesize the model, which helps to reduce modeling complexity. Experimental results indicate that this method can obtain accurate reconstructed 3D avatar models, and the running performance is faster than that of similar work. This research offers a useful tool for the manufacturers to quickly and economically create 3D avatars for products design, entertainment and online shopping.

## 1. Introduction

Modeling real humans in the virtual world is always a popular research topic since virtual avatars are not only essential for products design and manufacturing for living but also have a great deal of applications for games, movies, video conferences and online shopping. Reconstructing 3D avatars with accuracy, effectiveness and convenience is a topic of interest for researchers, in particular with the introduction of new hardware and technologies. Moreover, modeling is also expected to be straightforward and accessible to the common user. Recently, the success of low-cost RGB-D sensors such as Microsoft Kinect [1] has offered a promising means of 3D modeling, as current laser-based or image-based methods are either expensive or setting-complex [2]. The use of Kinect in object scanning, modeling and even environment mapping has led to explosive growth due to its economy and convenience [3].

As a portable depth camera, Microsoft Kinect consists of a color sensor and a depth sensor, which are able to obtain RGB images and depth images of objects within reachable range of vision. Based on the RGB-D data from Kinect, there has been a great deal of research on human reconstruction to achieve cost savings and greater efficiency [4,5,6,7,8,9], however, the final reconstructed human model is still far away to be acceptable. The existing research is primarily concerned with the following: (1) the use of fewer Kinects to increase modeling convenience and save on costs, as in [4,6,7,9]; (2) the use of fewer shape priors to reduce reconstruction complexity and obtain more of the actual body silhouette, such as in [5,8]; (3) the development of more sophisticated alignment algorithms to improve modeling accuracy, as in [5,7]; (4) the better quality RGB-D data inputs to obtain more model detail based on artificial denoising or a more reasonable capture strategy, as in [7,8,10,11,12]. Although previous works achieved a great deal in these aspects, it is still necessary to improve modeling quality and efficiency because of increasing demands on practicality.

In this paper, we take all these concerns into account and propose a new method for easy and fast 3D avatar reconstruction with an RGB-D sensor. A Kinect sensor is adopted to finish the data capture of the full human body, which preserves the benefits of convenience in practical applications without a complex setting. Users can easily run it at home or in an office with a personal computer and a small workspace. A turntable or second operator is not necessary during scanning. Furthermore, the accuracy of reconstructed models is better than that of the previous work in comparison. The contributions of this paper include the following:

(1) We adopt a new data capture strategy to scan the human body. It captures data from six views by rotating the human body, and in each view only three frames are obtained in upward, horizontal and downward directions (see details in Section 4.1). Thus, there are a total of 18 frames that cover the full human body, which dramatically reduces the amount of captured frames compared to previous work. For instance, in the system by Cui et al. [6], the user is turned around every 20 to 30 s to capture 10 frames within 0.5 s intervals; in the work of Tong et al. [5], each Kinect acquired images at 15 frames per second about every 30 s; in the system of Li et al. [7], the user was scanned from eight to nine views, and in each view, roughly 150 raw frames were captured. The greatly reduced amount of captured frames in our work helps reduce the computation loads for raw data analysis and thus speeds up the modeling procedure.
(2) We propose efficient alignment algorithms for aligning the point clouds in a single view and multi-views. Based on the new data capture strategy, new efforts have been made to achieve authentic aligned results quickly, including: (I) exploring the optimal combination of different correspondence pairing methods concerning alignment quality and efficiency both in the rigid and non-rigid alignment; (II) designing a three-step non-rigid alignment algorithm, which, unlike in previous works of human body modeling, introduces a pre-alignment of multi-view data frames using the model silhouettes to reduce the search space and thus improve alignment efficiency; (III) managing the point clouds throughout all the steps in alignment, which is helpful for generating more accurate results with no errors generated by the mesh operations; (IV) aligning the point clouds without the assistance of shape priors or subdivision tools, which helps to simplify the reconstruction procedure and reduce reconstruction complexity.
(3) We offer an easy-to-follow platform for 3D avatar modeling, one that highlights the technique details in each step and makes it possible for readers to follow along or develop such a method. With this platform, users can quickly generate 3D avatars for human factors/ergonomics applications, virtual entertainment and business.

New improvements have been made in the number of Kinects, scanning easiness and modeling efficiency. Because of these improvements, it is possible to reconstruct 3D avatars easily and quickly with an RGB-D sensor within a small workspace in home or office and obtain accurate results in most scanning scenarios. Experimental results are shown to validate the performance of our method and present its progress when compared with similar work.

## 2. Related Work

### 2.1. RGB-D Sensors for 3D modeling

Emerging RGB-D sensors integrate the strength of optical cameras and laser-based 3D scanners. They can obtain 3D measurement of objects and are almost portable like optical cameras. Thus, 3D modeling has become more practical with affordable RGB-D sensors, such as the Microsoft Kinect. Meanwhile, the open source point cloud library (PCL) offers direct accessibility to the Kinect camera [13]; RGB-D programs can be efficiently developed and closely integrated with the open platform OpenNI [14]. Microsoft also developed KinectFusion to help ordinary users scan and model a scene by hand-holding the Kinect camera. Many applications have been developed based on such a system; for example, Newcombe et al. used it for surface mapping and tracking [15], and Dong et al. applied it to reconstruct indoor space [16]. However, 3D modeling by KinectFusion is a depth-only approach and does not consider deformable shapes.

In 3D human reconstruction, the user cannot stand behind the 3D sensor and should be scanned from different views for data capture. In previous works, Weiss et al. adopted a single Kinect to capture humans wearing undergarments in various poses and computed body shapes by fitting them into a SCAPE model [2,4]. Tong et al. used three calibrated Kinects and a turntable to capture different body parts in the front and back sides and then reconstructed a 3D body shape [5]. Cui et al. reconstructed the human body with a system where the subject turned continuously around 360 degrees before a static Kinect while maintaining an approximate “T” pose [6]. Li et al. reported a 3D self-portrait system using a single Kinect by rotating the user based on 8~9 views with approximately 45 degrees between views [7]. Chen et al. realized 3D personalized avatar modeling with multiple Kinects targeting at a greater fast capture speed [8]. Zhu et al. proposed a dynamic human body modeling using a single RGB camera by taking advantage of human motion to decrease the chance of self-occlusion and utilized a general template to generate parametric human model [9]. However, a more ingenious approach for body scanning is still expected to improve modeling efficiency, accuracy and convenience. Our method proposes a new scanning strategy in 6 views and captures 18 frames to fully cover the body, which reduces the scan workload dramatically and thus improves modeling speed.

### 2.2. Multi-view Alignment

The 3D avatar is reconstructed by stitching together the sequence of captured RGB-D frames in different views. At first, it is important to execute rigid frame-frame alignment by matching their overlapping regions. Currently, both the image-based and shaped-based methods have been well studied [17]. Image-based alignment is typically achieved by sparse feature matching and epipolar geometry; Khoshelham et al. [18] presented an epipolar search method to obtain more accurate 3D correspondences. Shape-based alignment is mainly based on iterative closest point (ICP) algorithms, in which the algorithm proposed by Rusinkiewicz and Levoy [19] is preferred due to its efficiency and reliability. Since the ICP method and its variants can handle local rigid alignment well [20], most 3D reconstruction methods based on continuous depth frames have adopted such methods [21,22], in particular in human reconstruction with Kinects [6,7,8,23]. Given that calibration is required for the RGB images and depth images and geometric information is more reliable than visual information, we use the ICP-based method for local and global alignment, which helps to obtain more robust results in different cases.

In terms of aligning multi-view frames, common strategies include sequential alignment or pairwise alignment, which register each frame based on its previous frame. For instance, Chen and Medioni [24] proposed an approach to register consecutive frames with enough overlapping area without requiring point-to-point matching; Masuda [25] integrated the ICP algorithm and registered a depth image to a given model through a set of rigid motion parameters. However, it is possible to have tiny errors in pairwise alignment due to data noise and data incompleteness. These errors may accumulate when aligning multiple frames, particularly when thousands of frames are involved in the sequential approach. The accumulation of alignment errors leads to loop closure or even drift problems for long sequences of frames. The work of Henry el al. [26] and Endres et al. [27] addresses the loop closure problem well but relies on the distinctiveness of visual features, which requires the input data to have high resolution and little noise. To alleviate this issue, another strategy of simultaneous registration, namely, aligning all frames at once, was introduced; Li et al. [28] and Nishino and Ikeuchi [29] reported simultaneous registration methods. However, these approaches require search correspondences between the overlapping parts of all data frames over multiple views, which is exhaustive and computation-expensive. Considering alignment efficiency, we propose a three-step multi-view alignment algorithm for the sequence of point clouds in different views. It is useful for solving the loop closure problem and enables fast 3D avatar modeling.

Another problem in multi-view alignment is non-rigid deformation, which may be caused by inevitable relative motion or data loss during the data capture. In the literature, some researchers used prior templates to fit each scan frame for alignment [30,31]. This approach acquires a relative accurate template and markers for model fitting and is more common in dynamic human modeling. Some used semi-templates since an accurate template is difficult to obtain in many circumstances; for example, Li et al. [7] used a crude approximation of the scanned object as a shape hull, and Tong et al. [5] used a rough template constructed by the first data frame. In our method, because the new data capture strategy can obtain good quality data in most cases, we make use of the normal information of data points for pairwise correspondence computation between neighboring views. This attempt can reduce alignment complexity and improve the efficiency of 3D reconstruction under the condition of accuracy.

## 3. Overview

**Figure 1:**
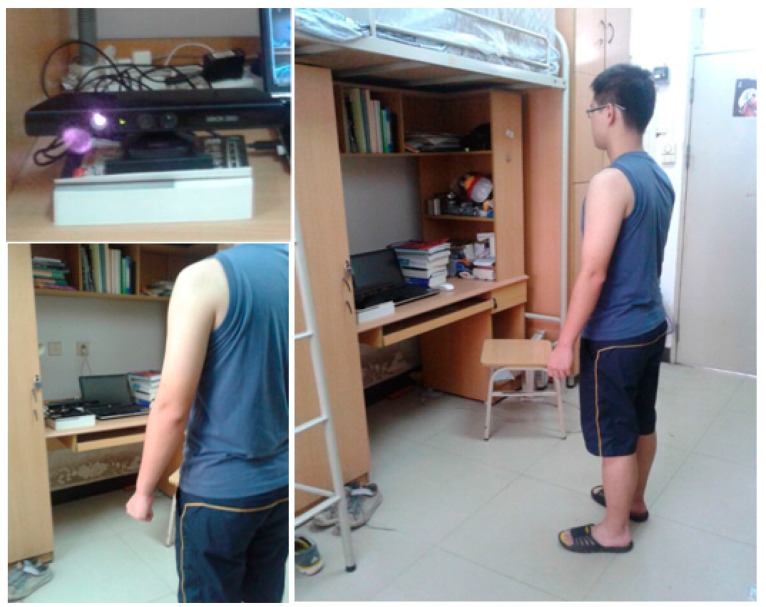
System setup of the 3D human body scanning scenario.

This method aims to provide an easy and fast approach of 3D avatar reconstruction to users. With a single RGB-D sensor such as Kinect, users can easily setup the scanning scenario and run the software on a personal computer to start and even finish the scanning and modeling procedure by themselves. The system setup is shown in Figure 1. The Kinect sensor is adjusted to the height of the waist of the user, and the scanning distance to the user is within 1 m. After starting the procedure, the user rotates himself/herself continuously to capture six views. In each view, the user is captured with three data frames automatically; namely, one frame is horizontal to the Kinect; one frame is 20 degrees above the horizontal; and one frame is 20 degrees below the horizontal. After the three frames are captured, a friendly voice reminds the user to move to the next view. The intervals for voice command is a controllable variable on the interface which the users can adjust for their preference.

**Figure 2:**
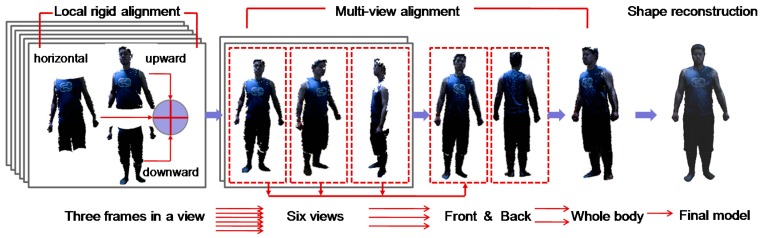
Pipeline of reconstruction of the 3D avatar.

When the 3D scanning is finished in all views, there are only 18 data frames that fully cover the human body. Thus, the computation speed for data denoising and alignment can be greatly enhanced due to the reduced number of frames. The reconstruction pipeline of our method is shown in Figure 2. The captured data frames are segmented from the background with specified depth thresholds and proceed with the necessary denoising of the data. Then, the three captured frames in a view are registered by local rigid alignment to fuse a view frame of the human body. Thereafter, 6 fused view frames are further globally aligned and merged. In the three-step multi-view alignment, the frames in neighboring views are first aligned and then gradually fused into two frames representing the body front and back sides; eventually, these two frames are stitched together to generate a complete data frame for the whole human body.

Based on this globally aligned data frame, a recent version of the Poisson reconstruction method [32] is adopted to generate a 3D human body model with a watertight surface. Due to the small amount of data frames in the local and global alignments, we can complete the reconstruction efficiently. Furthermore, the RGB data can be directly input into the Poisson reconstruction method; thus, the reconstructed model is mapped with texture at the same time.

## 4. Reconstruction Approach

### 4.1. Data Capture

**Figure 3:**
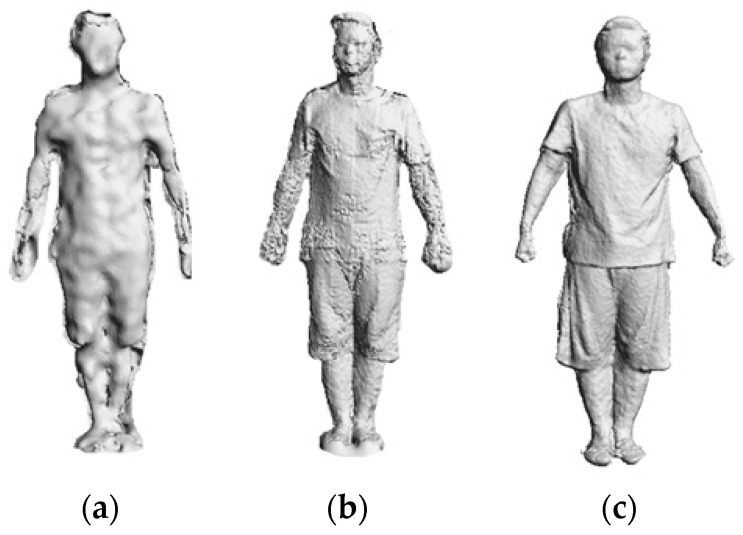
Scanned human bodies from different scanning distances: (**a**) 2 m distance; (**b**) 1.5 m distance; (**c**) 1 m distance.

The first step in 3D avatar reconstruction is to capture the RGB-D data. As discussed above, the user stands in front of the Kinect at a close distance (around 1 m, and 1.2 m is enough for the majority of people), which helps to obtain higher quality data. It is noted that the resolution of images from the Kinect is relatively low, in particular when the scanning distance increases, both the depth accuracy and resolution decreases quadratically. However, through experimental investigation, we find that the close scanning distance strategy can greatly improve the resolution of captured images. Figure 3 shows the difference in data quality of the scanned human body when adopting different distance strategies.

**Figure 4:**
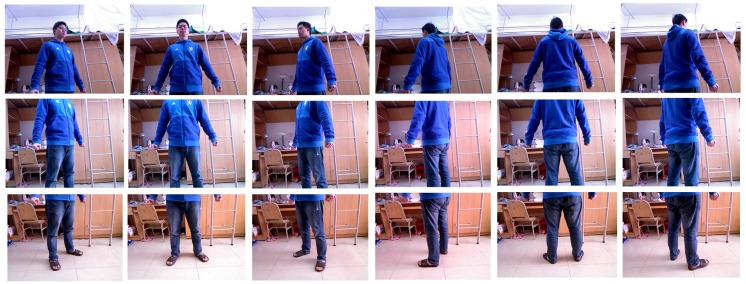
An example of 6 × 3 images of raw captured data.

Since the field of view of the Kinect is limited, we use three frames to capture the body in a single view. To realize an automatic capture, the Kinect’s motorized tilt feature is utilized to maneuver between the upward, horizontal and downward views. When the three frames have been captured successfully, the user rotates himself approximately 60 degrees. However, in practice, it is difficult to rotate precisely 60 degrees; thus, the user may rotate five times and evaluate based on perception, which has no influence on the results of reconstruction. That is because our three-step alignment algorithm can handle the rotation angle within 90 degrees. Figure 4 shows an example of the raw captured data of six views, which is a total of 18 frames of images.

During the scanning, a turntable or a second operator is not necessarily needed to assist with the procedure. Ordinary users with no domain knowledge can finish the data capture by themselves. Each data frame of RGB-D from the Kinect includes a 640 × 480 color image and a 640 × 480 depth image. By using OpenNI [14], 3D coordinates of the data are automatically generated, and the depth and color images are also calibrated by mapping the depth image to corresponding RGB points. The calibrated RGB-D images are then further segmented and denoised.

### 4.2. Segmentation and Denoising

The captured data frames cover not only the human body but also the surrounding environment in the scanning range. In order to segment the RGB-D data for the human body from the background, we first figure out the bottom of the human body data by cutting a plane, then give a threshold *θ* to the depth value (for the 1 m scanning distance, *θ* = 1.5 m) for separating the pixels since the scanned human body is close to the sensor.

**Figure 5:**
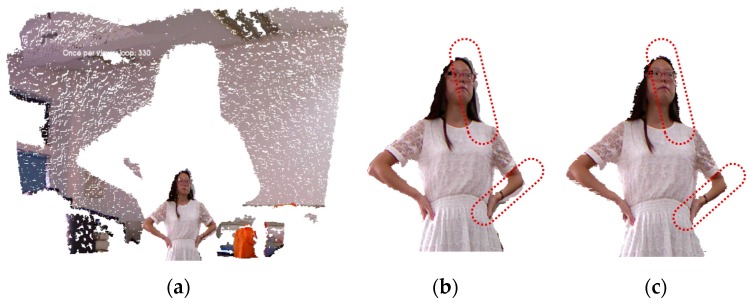
An example of captured data frames that is then removed from the background and denoised: (**a**) Captured raw data frame; (**b**) After removing background; (**c**) After denoising.

Meanwhile, the accuracy of the captured data can be affected by noise due to sensor coverage, lighting variations, surface transparency and scattering, and also by lateral noise, which is generated during registration of the RGB image and the depth image by OpenNI. For the RGB image, we use the work of Barron and Malik [33] in the reconstruction of shape, reflectance and illumination from a single image to smooth the errors caused by the lighting environment. As to the lateral noise, we draw out the edges of segmented data and then compare the edge pixels to compute the deviation of color brightness and hue. Here, we use the scan line to scan each row of pixels in the image for edge detection. Since the background has already been removed, the first pixel with color value is set as the edge of the human body, and the width of the edge is defined as *k* pixels (*k* = 10 in most cases). A comparison of the pixels starts from the first pixel on the edge until the last one. If the adjacent pixels on the edge line have deviation of more than 20% in color brightness or more than 3% in color hue, we delete the sequence of pixels before the pixel whose color value is quantitatively changed to remove the errors. For the depth image, due to the close scanning strategy adopted in this paper, the quality of depth values has been greatly improved as shown in Figure 3. First, pixels with zero depth value are deleted, and then the Laplacian method [34] is utilized to smooth the noise. A demonstration of data frame segmentation and denoising is shown in Figure 5.

After the segment and denoising process, the Kinect depth images are converted into 3D point cloud data before data alignment. In the following local and global alignment, we directly use the 3D point clouds to align different frames rather than the meshes generated by the data points. This attempt could help to improve alignment accuracy by avoiding the errors generated by mesh operations.

### 4.3. Local Rigid Alignment

For the three data frames in a single view, we implement pairwise local rigid alignment for consecutive frames by using the geometric ICP method. Although the ICP-based method may not perform well when there is small overlap between the aligned frames [8], it is not a bottleneck in this method due to the fact that there is enough overlap between two consecutive frames in a view that has only a 20 degree pitching angle between them. On the other hand, the limited number of data frames in a single view (only 3) leads to effective alignment in a short time, while in previous works, such as [8], the long sequence of frames usually needs to repeat the alignment in reverse to refine the alignment quality.

Denote $F=\{f_{i}^{k}|i=0,...,17,k=0,...,N_{f_{i}}\}$ as all the point clouds of the data frames, and $N_{f_{i}}$ is the number of points of frame $f_{i}$. Let $f_{i}$ be the horizontal frame, $f_{i+1}$ be the upward frame, and $f_{i+2}$ be the downward frame; first, $f_{i+1}$ and $f_{i}$, $f_{i+2}$ and $f_{i}$ are rigidly ICP-aligned, and then $f_{i}$, $f_{i+1}$, $f_{i+2}$ are further merged to generate the aligned frame in a single view, as shown in Figure 2. Similarly, the data frames captured in other views are rigidly aligned. There are a total of six merged data frames for the six scanning views.

For the point clouds in a single view, it is found that the means of the closest Euclidean distance works well in correspondence point searching and performs well in terms of alignment when compared with other methods (discussed later) such as the normal shooting method. Hence, we adopt the closet Euclidean distance as the metric to search the correspondence points for the input data points, and the data structure of *k*-*d* tree (*k* = 1) is adopted to speed up the searching process. The maximum search range for correspondence pairs is within the distance between the centers of gravity $\left(g_{i},g_{j}\right)$ of two point clouds $\left(f_{i},f_{j}\right)$, which is calculated as $d=sqrt({\left(g_{ix}-g_{jx}\right)}^{2}+{\left(g_{iz}-g_{jz}\right)}^{2})$ for the point clouds in a single view. Because the data frames in a view are captured on the horizontal, upward and downward view, the *y* values of data points, which indicate the height information, exhibit great differences in consecutive frames and thus are not incorporated into the gravity center calculation. With the searched correspondence points, the correspondence distance between the point pairs is calculated by the point-to-tangent plane method rather than the point-to-point method; namely, we calculated the distance between the input point and the tangent plane of its mate point.

Let $p_{i}$ and $q_{i}$ denote the correspondence point pairs of two consecutive point clouds; the correspondence distance of the point-to-tangent plane is calculated by:

(1) $$d(p_{i},q_{i})=(p_{i}-q_{i})\cdot n_{qi}$$

where $n_{qi}$ is the unit normal vector at points $q_{i}$. The normal of a point on a surface is approximated by estimating the normals of a plane tangent to the surface, which is transformed as a least-square plane fitting estimation problem in k-nearest neighboring points $Q^{k}$ [35]. Denote $\bar{q}$ as the centroid of $Q^{k}$ by $\bar{q}=\frac{1}{k}\sum_{i=1}^{k}q_{i}$; the normal of the point *q* is calculated by analyzing the eigenvalues and eigenvectors of the covariance matrix $C\in {\text{ℝ}}^{3\times 3}$ of $Q^{k}$ by:

(2) $$C=\frac{1}{k}\sum_{i=1}^{k}\left(q_{i}-\bar{q}\right){\left(q_{i}-\bar{q}\right)}^{T},C{\bar{v}}_{j}=\lambda_{j}{\bar{v}}_{j},j=\{0,1,2\}$$

where *C* is positive semi-definite and symmetric, and its eigenvalues are $\lambda_{j}$. When $0\leq \lambda_{0}\leq \lambda_{1}\leq \lambda_{2}$, the eigenvector corresponding to the smallest eigenvalue is the approximation of the inquired normal vector. Thus, the alignment of two consecutive frames is to minimize the distance by solving the error function:

(3) $$E=\underset{M}{\arg\min}\sum_{i}{\left\|\left(Rp_{i}+T-q_{i}\right)\cdot n_{qi}\right\|}^{2}$$

The solution of Equation (3) can be obtained by approximating the non-linear least-squares method into an optimized linear least-squares method [36]. $p_{i}$ and $q_{i}$ are represented as $p_{i}={\left(p_{ix},p_{iy},p_{iz},1\right)}^{T}$ and $q_{i}={\left(q_{ix},q_{iy},q_{iz},1\right)}^{T}$, and *M* is a 4×4 3D rigid-body transformation matrix, which is composed of a rotation matrix R(α,β,γ) and a translation matrix $T(t_{x},t_{y},t_{z})$, where

(4) $$T(t_{x},t_{y},t_{z})=\left[\begin{array}{cccc} 1 & 0 & 0 & t_{x}\\ 0 & 1 & 0 & t_{y}\\ 0 & 0 & 1 & t_{z}\\ 0 & 0 & 0 & 1 \end{array}\right],R(\alpha ,\beta ,\gamma )=\left[\begin{array}{cccc} r_{11} & r_{12} & r_{13} & 0\\ r_{21} & r_{22} & r_{23} & 0\\ r_{31} & r_{32} & r_{33} & 0\\ 0 & 0 & 0 & 1 \end{array}\right]$$

with $r_{11}=\cos\gamma \cos\beta ,r_{12}=-\sin\gamma \cos\alpha +\cos\gamma \sin\beta \sin\alpha ,r_{13}=\sin\gamma \sin\alpha +\cos\gamma \sin\beta \cos\alpha$, $r_{21}=\sin\gamma \cos\beta ,r_{22}=\cos\gamma \cos\alpha +\sin\gamma \sin\beta \sin\alpha ,r_{23}=-\cos\gamma \sin\alpha +\sin\gamma \sin\beta \cos\alpha$, $r_{31}=-\sin\beta ,r_{32}=\cos\beta \sin\alpha ,r_{33}=\cos\beta \cos\alpha$, α,β,γ are the rotation radians about the *x*, *y* and *z* axis in the right hand coordinate respectively, namely, $R_{z}\left(\gamma \right)\cdot R_{y}\left(\beta \right)\cdot R_{x}\left(\alpha \right)=\left[\begin{array}{ccc} \cos\gamma & -\sin\gamma & 0\\ \sin\gamma & \cos\gamma & 0\\ 0 & 0 & 1 \end{array}\right]\cdot \left[\begin{array}{ccc} \cos\beta & 0 & \sin\beta \\ 0 & 1 & 0\\ -\sin\beta & 0 & \cos\beta \end{array}\right]\cdot \left[\begin{array}{ccc} 1 & 0 & 0\\ 0 & \cos\alpha & -\sin\alpha \\ 0 & \sin\alpha & \cos\alpha \end{array}\right]$.

Suppose ∀θ≈0, then sinθ=0; thus, when α,β,γ≈0, *M* can be approximated as:

(5) $$M=T(t_{x},t_{y},t_{z})\cdot R(\alpha ,\beta ,\gamma )=\left[\begin{array}{cccc} 1 & -\gamma & \beta & t_{x}\\ \gamma & 1 & -\alpha & t_{y}\\ -\beta & \partial & 1 & t_{z}\\ 0 & 0 & 0 & 1 \end{array}\right]$$

Then, Equation (3) can be solved by linear expression as:

(6) $$\left(Rp_{i}+T-q_{i}\right)\cdot n_{i}=\left(M\left(\begin{array}{c} p_{ix}\\ p_{iy}\\ p_{iz}\\ 1 \end{array}\right)-\left(\begin{array}{c} q_{ix}\\ q_{iy}\\ q_{iz}\\ 1 \end{array}\right)\right)\cdot \left(\begin{array}{c} n_{qix}\\ n_{qiy}\\ n_{qiz}\\ 0 \end{array}\right)$$

It is noted that the approximation in the solution is based on the assumption of α,β,γ≈0, and the results will become more accurate when the two aligned point clouds are closer after iterative computation. In our scanning strategy, since the consecutive frames in a view are already very close after the 20 degree rotation, this solution works very well for the local alignment. The criterion for ending the computation iteration is when $(\left|M^{k+1}\right|-\left|M^{k}\right|)<0.01$ between two steps. However, there are usually less than 10 computation iterations since the point-to-tangent plane method converges quickly.

**Figure 6:**
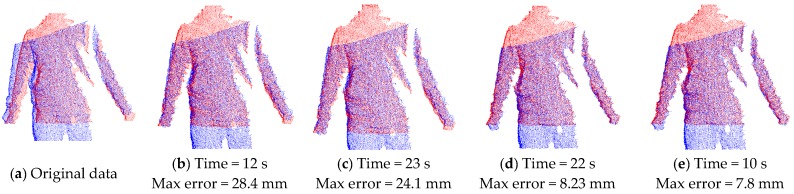
Local rigid alignment results when using different methods in correspondence pairing computation: (**a**) Original two consecutive point clouds in a view (red and blue); (**b**) Result by using CED and PTP; (**c**) Result by using NS and PTP; (**d**) Result by using NS and PTTP; (**e**) Result by using CED and PTTP. * CED: closest Euclidean distance method in correspondence point searching; NS: normal shooting method in correspondence point searching; PTP: point-to-point method in correspondence distance calculation; PTTP: point-to-tangent plane method in correspondence distance calculation.

As discussed above, there are alternative methods in correspondence point searching and correspondence distance calculation. We illustrate the result of the method used in this paper and compare its performance with that using the combination of other methods. As shown in Figure 6, Figure 6a is the input of two original point clouds in a single view (the red one and blue one), Figure 6b–e are the aligned results when employing different methods on the same two input data. It can be seen that Figure 6b,e using closest Euclidean distance (CED) in correspondence point searching have faster computation speed than those using the normal shooting (NS) method, while Figure 6d,e, which use the point-to-tangent plane (PTTP) method in correspondence distance calculation, have much better alignment results with smaller maximum error of Euler distance between two aligned frames. Such a comparison shows that the closest Euclidean distance and point-to-tangent plane methods used in this paper can quickly achieve local rigid alignment with good results.

With the rigid alignment algorithm, the three data clouds in a single view are aligned by the group of two consecutive frames, and then they are merged into one point cloud for this view. Because point density increases greatly after merging the three frames, a down-sampling action is undertaken using the voxel grid of PCL, in which the space is divided into a set of tiny 3D boxes and all the points present in space are approximated with their centroid.

### 4.4. Multi-View Alignment

After all the data frames have been rigidly aligned group by group and the data frames for the 6 views are generated, a three-step multi-view alignment is proposed to further globally align and fuse them together. As reviewed in Section 2, the multi-view alignment could be simultaneous, sequential or pairwise registration. However, simultaneous registration requires more computation iterations to converge, whiles sequential registration is dependent on previous frames and accumulates registration errors easily. To achieve both alignment efficiency and accuracy, the multi-view alignment is designed, which includes three steps: (1) pre-alignment of the multi-view frames; (2) rigid alignment of the multi-view frames and (3) non-rigid alignment of the multi-view frames again. Pairwise alignment is performed between neighboring views, and the geometric constraint is used for the computation of pairwise correspondence to achieve global alignment of multiple views.

**Figure 7:**
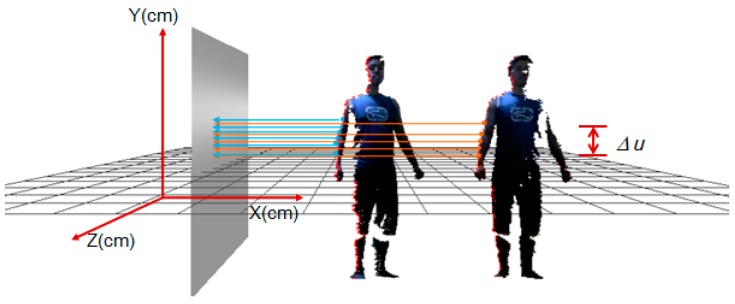
Example of searching correspondence points on the silhouette.

Denote $S=\left\{s_{j}^{k}|j=0,...,5,k=0,...,N_{s_{j}}\right\}$ as the merged point clouds of the data frames for the 6 views, and $N_{s_{j}}$ is the number of points of the frame $s_{j}$. Let *s*_0_, *s*_1_, *s*_2_ denote the frames on the front side, the left-to-front side and the right-to-front side, respectively, and let *s*_3_, *s*_4_, *s*_5_ denote the frames on the back side, the left-to-back side and the right-to-back side, respectively. Following are the specific algorithms in the three steps:

*Step 1: Pre-alignment of the multi-view frames*. To avoid an exhaustive search for all possible correspondence pairs, thus improving alignment efficiency, we first perform a pre-alignment of all the partial views by searching their pairwise correspondence on the silhouette. Considering that the extraction of the body's silhouette is easy to confine to local sharp areas, such as the fingers, we determine the silhouette of the point cloud in a differential way to achieve global smooth distribution. Set ∆*u* (∆*u* = 10 mm) as a differential unit in the 2D space of the y axis, and then the number of differential units $N_{d}$ is decided by the maximum and minimum of the point cloud on the *y* axis, namely, $N_{d}=(y_{\max}-y_{\min})/\Delta u$; then, the 3D space is divided into $N_{d}$ sub-regions accordingly. Given a point $p_{0}=(p_{0x},p_{0y},p_{0z})$, which is located in the ith ($i=(p_{0y}-y_{\min})/\Delta u$) sub-region, we designate a project plane vertical to XOZ at the inquired side of the point cloud, then search all the points $\left\{p_{m}\right\}$ in the *i*th sub-region and calculate their distances to the plane by:

(7) $$d_{m}(p_{m},\theta_{t})=p_{mx}\sin\theta_{t}-p_{mz}\cos\theta_{t}$$

where $\theta_{t}$ denotes the angle between the project plane and *y* axis. Suppose $\forall \theta_{t}=\{-120^{{}^{\circ}},\;-90^{{}^{\circ}},\;-60^{{}^{\circ}},\;60^{{}^{\circ}},\;90^{{}^{\circ}},\;120^{{}^{\circ}}\}|t=0,...,5$, and $\theta_{t}$ is the same for all the points in the same view. We sort all the points in a sub-region by the descending distance $d_{m}$ and choose *k* (*k* = 5) points with minimal values as the correspondence points on the silhouette. Figure 7 shows an example of the searching correspondence points of the silhouette for two neighboring views. With the searched silhouette points in the neighboring views, those with the same descending orders in the same number of sub-regions are chosen as correspondence pairs.

**Figure 8:**
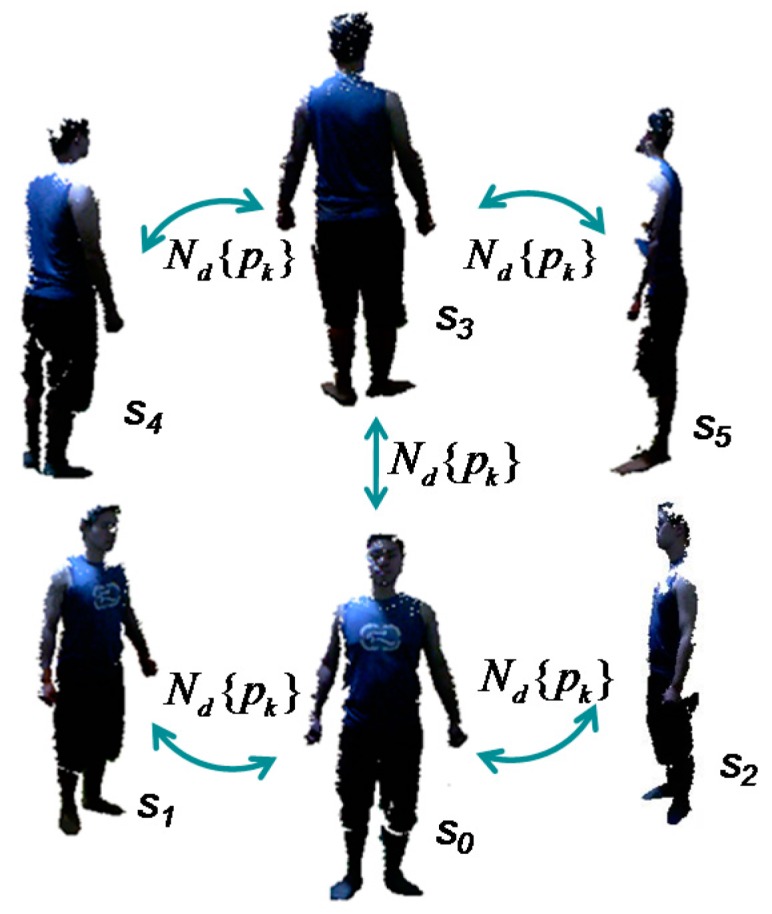
Pre-alignment between the 6 partial views.

When all the partial views finish searching and paring the correspondence points on the silhouette, the neighboring views can be aligned by pairwise correspondence based on the closest Euclidean distance. The pre-alignment procedure for all 6 views is repeated 5 times, as shown in Figure 8. In theory, pre-alignment can be conducted iteratively in turn, such as by clockwise order. However, the scanned body has more exposure to the sensor on the front and back sides; thus, the captured frames on these two sides have more data points, which may offer more overlap between neighboring views. This can help to improve the quality of alignment and obtain more accurate results. Therefore, pre-alignment is first conducted for the front and back views with the side views, namely, *s*_0_ and *s*_1_, *s*_0_ and *s*_2_, *s*_3_ and *s*_4_, *s*_3_ and *s*_5_, and finally *s*_0_ and *s*_3_. In the reference, similarly, Wang et al. [34] had used the contours coherence to align two wide baseline range scans with limited overlap.

**Figure 9:**
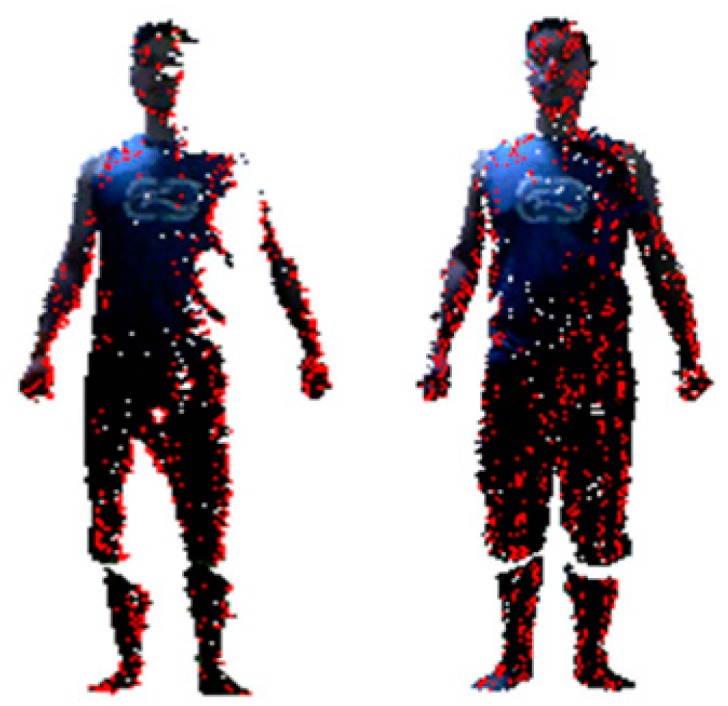
Calculated correspondence points of two frames in neighboring views.

*Step 2: Rigid alignment of the multi-view frames*. With pre-alignment, the partial views already have optimal initial alignment. They can be further rigidly aligned by the group of two point clouds in neighboring views to obtain a more accurate body shape in the 3D space. In this step, we use the ICP-based method again as discussed in Section 4.3, namely, combining the closest Euclidean distance method in correspondence point searching and the point-to-tangent plane method in correspondence distance calculation. The maximum range of correspondence point searching is still the distance between the centers of gravity of two point clouds $\left(g_{i},g_{j}\right)$; however, its calculation is updated as $d=sqrt({\left(g_{ix}-g_{jx}\right)}^{2}+{\left(g_{iy}-g_{jy}\right)}^{2}+{\left(g_{iz}-g_{jz}\right)}^{2})$ for the point clouds of partial views. Our experimental investigation finds that the correspondence pairs that have too much distance between them lead to inaccurate correspondence and should therefore be discarded. Figure 9 demonstrates the calculated correspondence points of two frames in neighboring views. As mentioned in Section 4.3, the rigid alignment solution is based on the assumptions of α,β,γ≈0, and the two point clouds are close to each other. In this step, the pre-alignment in Step 1 has already determined a close initialization for the neighboring data frames, which can ensure that the alignment solution works well. The pairwise rigid alignment procedure for all six views is similar to that shown in Figure 8. There are five times alignments in total for the front view, the back view and their neighboring views on the left and right sides.

**Figure 10:**
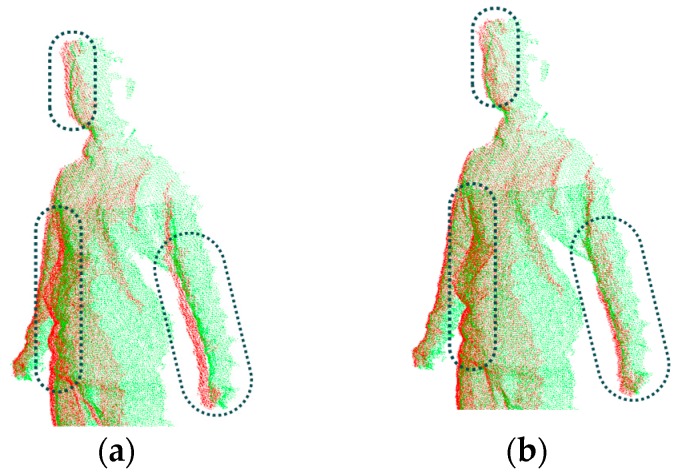
The loop closure problem from point clouds in rigid alignment: (**a**) Before non-rigid alignment; (**b**) After non-rigid alignment. It can be seen from the comparison on the marked areas.

*Step 3: Non-rigid alignment of the multi-view frames.* Loop closure is an important problem in alignment, which means after multiple times of rigid alignment, the shape of the aligned object has no tight closure due to the accumulation of errors occurred in every rigid alignment. Although the above two steps have already achieved primary registration for the multi-view frames, when we merge them progressively, the loop closure problem occurs inevitably due to the possible relative motion (as shown in Figure 10). It is necessary to solve this problem by distributing the errors evenly over all the sequential frames, which is rather effective for the static objects. In practice, during the scanning process, the human body does not remain completely still and inevitably exhibits movement. Meanwhile, the deformation is also possibly caused by the dress, hairstyle or even the calibration of the sensors. Thus, a non-rigid global alignment is required to deal with such problems.

Similar to local rigid alignment, we first compute pairwise correspondences between the neighboring views and then register and merge all the partial views progressively into a final global frame of the human body. Given that adding higher-order information to the minimization criterion, such as normals and curvatures, can decrease the possibility of convergence to local minima in a non-rigid situation, we take into consideration the normals of the point cloud in the stage that involves searching pairwise correspondence points. Namely, the normal shooting method is used to search for the correspondence points, where the intersecting points between the normal vector of the input point and the target point cloud are selected as the candidate points. The data structure of *k*-*d* tree is also utilized to make the searching faster, where *k* = 10 candidate points nearest to the input point are searched to compare their distances to the normal vector. In the set of candidate points, the one having the shortest distance is selected as the mate point. The maximum search range for correspondence pairs is still set as the distance between the centers of gravity of two point clouds. In the stage of correspondence distance calculation, the point-to-tangent plane method, which contributes substantially to good alignment results, is adopted. Later, we also compare the performance of non-rigid alignment when employing different methods for correspondence point searching and correspondence distance calculation.

Once computing the pairwise correspondences between the point clouds of neighboring views, we construct the deformation model in the form of a deformation graph to allow natural shape deformations. For the two point clouds p,q, which will be aligned, p is first down-sampled at the rate of 50 mm, where each point is modeled as a node in the graph, and then p,q are down-sampled at the rate of 15 mm to establish correspondence pairs. An affine transformation matrix including a 3 × 3 rotation matrix *R* and a 3 × 1 translation vector t is associated to each graph node. Furthermore, each graph node $x_{i}$ influences a deformation within the surrounding space of radius $r_{i}$. Denote $\left(R_{i},t_{i}\right)$ as the transformation matrix of graph node $x_{i}$; a vertex $v_{j}$ of the embedded shape will be transformed by:

(8) $$v_{j}^{\prime }=\sum_{x_{i}}\bar{w}(v_{j},x_{i},r_{i})[R_{i}(v_{j}-x_{i})+x_{i}+t_{i}]$$

where $\bar{w}$ is the normalized weights and $\bar{w}(v_{j},x_{i},r_{i})=\max(0,{\left(1-d^{2}(v_{j}-x_{i})/r_{i}^{2}\right)}^{3})$; $d(v_{j}-x_{i})$ is the distance from $v_{j}$ to $x_{i}$; when the distance exceeds the influence space of radius $r_{i}$($r_{i}=50\;\mathrm{mm}$), the weight is 0. During the transformation, to avoid looking through all the graph nodes and to speed up the procedure, the *k*-*d* tree is further used to search out the nodes within the space of radius $r_{i}$.

In theory, the goal of non-rigid alignment is to determine the affine transformation of the source point cloud to the target one with minimal energy [28], thus we formulate non-rigid alignment as the minimization of the energy:

(9) $$E=\arg\min\;(w_{1}E_{rigid}+w_{2}E_{smooth}+w_{3}E_{cor})$$

where $E_{rigid}$ and $E_{smooth}$ serve to ensure the local rigidity and smoothness of deformation, respectively, and $E_{cor}$ measures the deviation of the correspondence. For two graph nodes $\left(x_{i},x_{j}\right)$, $E_{smooth}={\sum_{x_{i}}\sum_{x_{j}}\bar{w}(x_{j},x_{i},r_{i}+r_{j})\left\|R_{i}(p_{j}-p_{i})+p_{i}+t_{i}-(p_{j}+t_{j})\right\|}_{2}^{2}$, $E_{rigid}$ penalizes the deviation of each transformation from a pure rigid motion and $E_{rigid}=\sum_{i}Rot(R_{i})$. Suppose *e*_1_, *e*_2_, and *e*_3_ are the 3 × 1 column vectors of the 3 × 3 matrix R; then, Rot(R) = ${\left(e_{1}^{t}\cdot e_{2}\right)}^{2}+{\left(e_{1}^{t}\cdot e_{3}\right)}^{2}+{\left(e_{2}^{t}\cdot e_{3}\right)}^{2}+{\left(1-e_{1}^{t}\cdot e_{1}\right)}^{2}+{\left(1-e_{2}^{t}\cdot e_{2}\right)}^{2}+{\left(1-e_{3}^{t}\cdot e_{3}\right)}^{2}$. Let *C* be the set of all pairwise correspondence, and *c*_1_ and *c*_2_ are the correspondence pairs for each correspondence, we therefore adopt the point-to-tangent plane method in correspondence distance calculation, thus, $E_{cor}$ is updated as $E_{cor}=\frac{1}{\left|C\right|}{\sum_{(c_{1},c_{2})\in C}\left\|\left(\left(\sum_{x_{i}}\bar{w}(c_{1},x_{i},r_{i})(R_{i}(c_{1}-x_{i})+x_{i}+t)\right)-c_{2}\right)\cdot n_{c_{2}}\right\|}^{2}$.

The optimization of Equation (9) can be solved by using the Nonlinear Least Squares algorithm in [37]. Equation (9) is minimized by Gauss-Newton iterations, and a sparse Cholesky factorization is employed to solve the linear equation in each iteration. We follow the findings in [8] and set the weight parameters as $w_{1}=500,w_{2}=2.0,w_{3}=2.5$. The description of the non-rigid alignment algorithm for two point clouds in neighboring views is shown in Algorithm 1. Generally, the solver of Gauss-Newton can converge within 15 iterations. During this non-rigid alignment, the most important factor influencing the computation is from the correspondence point pairing. If given some initial registration error, it will lead to repeated matching dozens of times. Fortunately, in the first step of pre-alignment, all the partial views have already been configured well with initial alignment; thus, this non-rigid multi-view alignment is fast to converge.
**Algorithm**
**1:** Non-rigid alignment for two point clouds in neighboring views.1:point cloud Q:= down-sampled P1 (50 mm)2:point cloud S1:= down-sampled P1 (15 mm), point cloud S2 := down-sampled P2 (15 mm)3:new embedded deformation graph G with G.node_amount = Q.point_amount4:for i:= 1 to Q.point_amount5:   G.node[i].xyz := Q.point[i].xyz6:   G.node[i].translation_matrix:= 3 × 1 zero matrix7:   G.node[i].rotation_matrix:= 3 × 3 identity matrix8:end for9:while not reach max iteration times or stop condition10:for i:= 1 to S1.point_amount11:   S1.point[i].correspondence:= corresponding point in S212:end for13:matrix X:= all rotation matrix and translation matrix in G14:new Gauss-Newton iterator (X = arg min ($w_{1}E_{rigid}+w_{2}E_{smooth}+w_{3}E_{cor}$))15:new matrix f16:while not satisfy precision requirement17:   compute matrix f according to $w_{1}E_{rigid}+w_{2}E_{smooth}+w_{3}E_{cor}$   f^T^f = $2(w_{1}E_{rigid}+w_{2}E_{smooth}+w_{3}E_{cor})$18:   matrix J := jacobian_matrix(f)19:   Cholesky_Decompositon.solve_linear_equation   Obtain the iteration $h_{k+1}=h_{k}+\Delta h$ where J^T^J Δ*h_k_* = −J^T^f20:   X:= X + *h*21:   Update E;22:end while23:deform P1 with G24:end while

**Figure 11:**
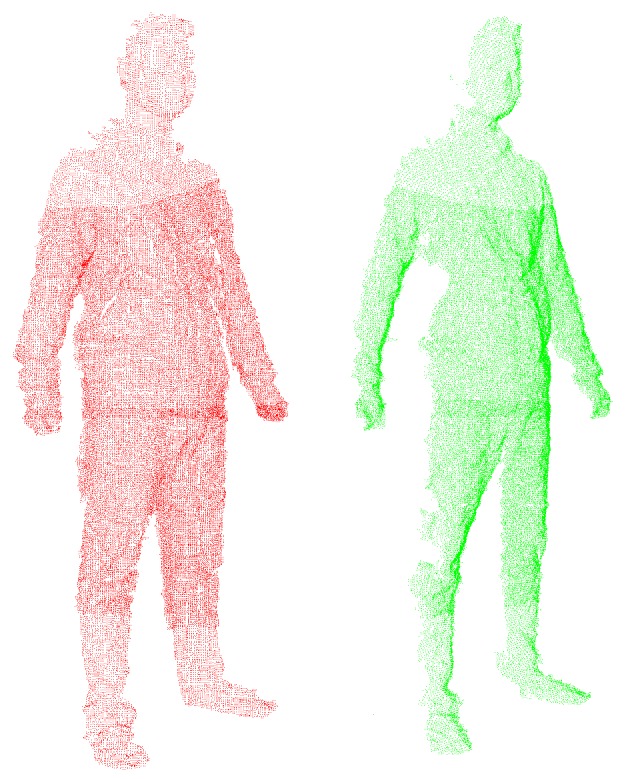
Two point clouds of neighboring views for non-rigid alignment.

**Figure 12:**
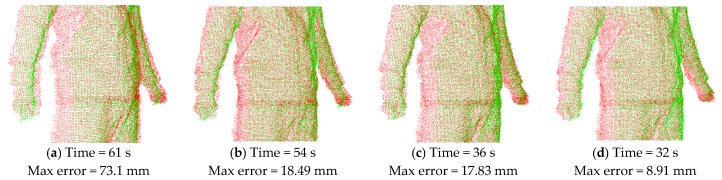
Non-rigid alignment results when using different methods in correspondence pairing computation: (**a**) Result by using CED and PTP; (**b**) Result by using NS and PTP; (**c**) Result by using CED and PTTP; (**d**) Result by using NS and PTTP. * CED: closest Euclidean distance method in correspondence point searching; NS: normal shooting method in correspondence point searching; PTP: point-to-point method in correspondence distance calculation; PTTP: point-to-tangent plane method in correspondence distance calculation.

To demonstrate alignment performance using the normal shooting and point-to-tangent plane methods in correspondence computation in the above, we compare the results of non-rigid alignment on the same input of two point clouds in neighboring views (as shown in Figure 11) when adopting various combinations of methods in correspondence pairing computation. As shown in Figure 12, the results from the point-to-tangent plane (PTTP) method indicated in Figure 12c,d have better non-rigid alignment quality which have smaller maximum error of Euler distance between two aligned frames and much faster speed than those based on the point-to-point (PTP) method indicated in Figure 12a,b. Thus, the point-to-tangent plane method works well for no-rigid alignment. Meanwhile, a comparison of the results of Figure 12a–d for closest Euclidean distance (CED) and normal shooting (NS) methods in correspondence point searching shows that the normal shooting (NS) method can improve both alignment efficiency and quality. However, in local rigid alignment, it is observed that the NS method contributes to inferior alignment performance. This phenomenon is due to the fact that the NS method requires more time in correspondence point searching and may obtain fewer points but better accuracy than the CED method. The number of correspondence points influences the subsequent steps in non-rigid alignment and may lead to remarkable differences in the total performance of alignment, which is more significant than the difference in correspondence point searching. However, the number of correspondence points has little influence on subsequent steps in local rigid alignment, the final performance of which is mainly affected by the time involved in correspondence point searching. Meanwhile, the point clouds have been down-sampled before the non-rigid alignment step, and the number of data points is greatly reduced, while the point clouds are much denser in the local rigid alignment. These facts cause the difference in performance of the NS and CED methods in local rigid alignment and global non-rigid alignment, respectively.

With the deformation model, non-rigid alignment is executed iteratively for all partial views following the procedure in Figure 8. The difference with Step 1 and Step 2 above is that it aligns and merges the two neighboring views into a single metaview in a progressive way. Namely, *s*_0_ and *s*_1_ are aligned, *s*_0_ and *s*_2_ are aligned, and then *s*_1_ and *s*_2_ are merged with *s*_0_ into a metaview *t*_0_. Similarly, the frames of *s*_3_, *s*_4_, *s*_5_ are aligned between neighboring views and merged into a metaview *t*_1_, Finally, the two metaviews *t*_0_ and *t*_1_ are aligned and merged into a point cloud for the global view of the human body. To avoid non-uniform sample density, the merged data frame is followed with a down-sampling operation with the rate of 2 mm.

### 4.5. Watertight Reconstruction

After all the point clouds are globally aligned and merged into a data frame for the whole human body, we create a watertight mesh surface of the human body by using a recent version of screened Poisson reconstruction method [32]. The point cloud is first transferred into the polygon format, which contains RGB data together, and then delivered to the surface reconstruction method. Since we deal with the point clouds throughout all the steps, it is helpful to generate a more accurate surface without errors being generated by the mesh operations. Furthermore, due to the aligned point cloud having full coverage of the scanned human body, we do not use shape priors or hulls to fill in the data loss regions (holes) or apply subdivision tools such as quadratic Bézier curves to interpolate the depth data. Such new attempts help to simplify the reconstruction procedure and reduce the complexity of 3D human reconstruction. Another new feature of reconstruction is that the RGB data are associated with the depth data and can be directly mapped on the generated surface; furthermore, the generated visualization is acceptable for most applications of CAD-based design and virtual shopping. In such cases, the reconstructed avatars are further extracted for accurate anthropometric data/features, which will be used in human factor/ergonomic applications to ensure that the designs and standards are realistic. This evolved function can save steps and reduce time spent on calculating and mapping the texture.

It is noted that there may be data holes in the final point cloud for the whole human body, for example, the hole on the head that cannot be reached by the sensor and the hole on the body caused by self-occlusion. The screened Poisson reconstruction method can automatically fill in the holes during mesh generation. However, it occasionally leads to obvious artifacts when there are big holes in the point cloud.

### 4.6. Texture Mapping

Texture mapping for reconstructed 3D human models is optional in this method in case a more refined image of the reconstructed models is preferred for the final presentation of the models, such as 3D portrait. In order to map the texture information of RGB images to the corresponding 3D points on the reconstructed surface, a closed mesh of the 3D models is first constructed by segmenting and unfolding the 3D surface to the 2D domain. Then, the 18 frames of the cloud points of the human body, which have been assigned with colors through Kinect’s calibration of RGB images and depth images, are aligned without down-sampling by the local and global alignment algorithms. For a 3D point p on the mesh surface, its texture information is assigned with the weighted average of color values of the points in the data frames which are near *p* under a threshold.

## 5. Results and Discussion

In this section, we demonstrate the reconstruction results for 3D avatars based on our method in different scanning cases and compare them with the results of similar works. The limitations of the approach are also discussed. All the RGB-D datasets in the scanning experiments are captured with a single Microsoft Kinect facilitated with a notebook computer (CPU Intel i5-2450 m 2.5 GHz, 4 GB RAM) in a living room. The experiments are performed easily with the aid of an instructional voice that reminds the subject to rotate, and they can be executed without turntables or other operators.

### 5.1. Experimental Results

**Figure 13:**
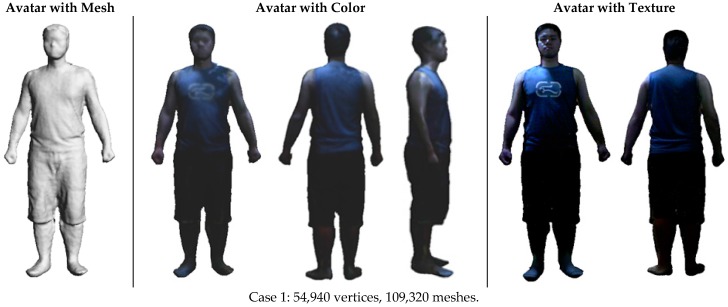

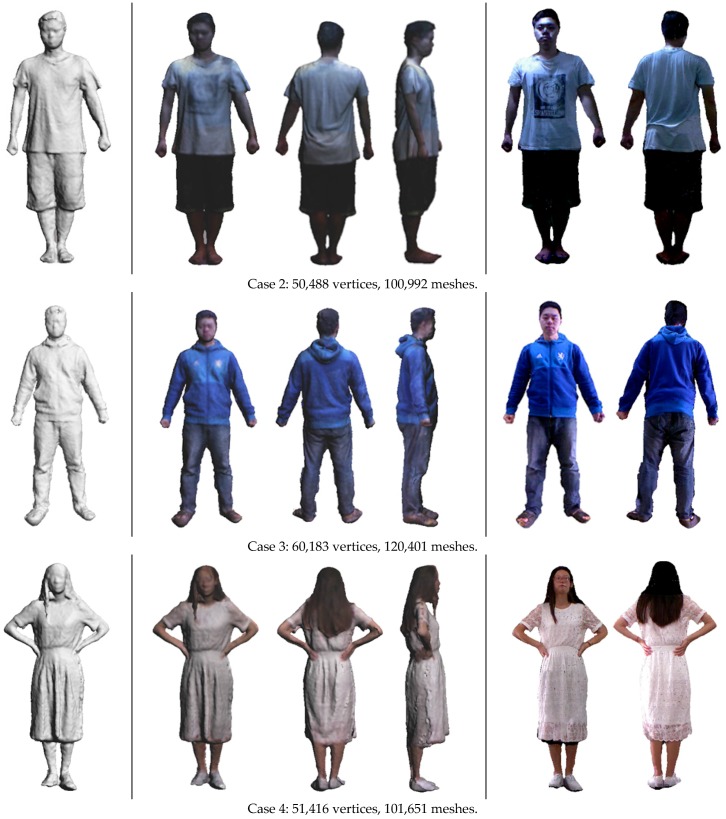
Reconstruction results of 3D avatars in four scanning cases.

Although there are a variety of human scanning scenarios with different specifications, we choose five cases that represent classic scenarios in practice, including a male subject wearing tight clothing (Case 1), loose clothing (Case 2) and winter clothing (Case 3), as well as a female subject wearing a piece of floppy skirt (Case 4). These cases involve possible relative body motion and deformation at different levels. The cases of a male subject wearing loose and winter clothing have more deformation than the cases of a male subject wearing tight clothing. The case of the female subject is the most challenging because she not only wears a piece of floppy skirt, which deforms easily and swings, but she also has fluttering long hair. In addition, she is posed with arms akimbo, which could cause self-occlusion during scanning. Following the reported reconstruction pipeline, the 3D avatar models in these cases are reconstructed in a user-friendly and efficient way. Figure 13 shows the reconstruction results, which include reconstructed avatar with mesh, avatar with color and avatar with texture. It can be seen that our method can achieve desirable results of 3D avatars, with realistic clothing wrinkles and deformation as well as realistic hairstyles in the case of the female subject. Compared to the current methods based on laser scanning or infrared scanning, the Kinect scanning method is quite flexible and able to conveniently obtain the virtual avatars of users with low cost.

### 5.2. Performance Comparison

To further demonstrate the performance of our method, the biometric measurement of reconstructed virtual avatars in the above experimental cases is calculated, and such measurement is conducted on the real bodies. Table 1 shows the average errors of biometric measurement in centimeters between the calculated and measured results of the human bodies. Meanwhile, we compare the similar results reported in [5,8] using two Kinects, and those reported in [6,9] using one Kinect. It can be seen that our results are better than the previous results on most of the items, such as the neck to hip distance, waist length, hip length, arm length and leg length. Although the biometric errors are not absolute when employing different human datasets, the 3D modeling method should be able to work for different subjects and has no obvious influence by the subjects’ appearance, such as clothing and accessories. The comparison results in Table 1 indicate that our method is capable of obtaining accurate reconstructed human models.

We also evaluate the running time of the thorough scanning and modeling procedure to validate the performance of this method. Table 2 shows the average running time spent during each step and also the computation cost of each step reported in [6,9], both of which used one Kinect. It can be seen that the configuration of the hardware in our method is much simpler than those in [6,9], and our method requires almost 40% less time than the other solutions, which did not include the time for data scanning, as in [6]. The comparison demonstrates that our method has made much progress in running performance since we have designed a more concise data capture strategy and more efficient alignment algorithms.

Though this proposed method can reconstruct 3D avatars easily and fast with desirable results in most cases, however, if the poses exhibit substantial self-occlusion, such as crossed hands and crouching, we find that the data incompleteness caused by self-occlusion will lead to artifact in the results. However, in practice, the scanning poses with substantial occlusion may be deliberate in just a few applications, such as 3D portraits print.

## 6. Conclusions and Future Work

In this paper, we propose an easy and fast method of 3D avatar reconstruction with an RGB-D sensor. The user can easily implement the scanning and modeling procedure with the help of a personal computer and an RGB-D sensor in home or offices. During the scanning process, there is no need for a turntable or a second operator, and ordinary users with no domain knowledge can finish the data capture by themselves. The contributions of this method include a new data capture strategy that captures 18 frames in 6 views to fully cover the human body and efforts in proposing efficient alignment algorithms to quickly achieve a globally aligned point cloud for the entire human body. Furthermore, we do not use shape priors or subdivision tools to assist the alignment and reconstruction, which reduces the complexity of the modeling procedure. A comparison of the reconstruction results shows that this method can obtain reconstructed 3D avatars in various situations, and the accuracy and running performance is superior to that of similar works. This reconstruction method is meaningful for products design and digital entertainment. The direction of future research include increasing modeling robustness when there is substantial occlusion or deformation of the scanned body and improving the quality of scanned data to allow for finer detail and more realistic texture. The former may require a more sophisticated data capture strategy and alignment algorithms; the latter could be solved by more advanced denoising algorithms and higher resolution RGB-D sensors.

## Figures and Tables

**Table 1:** Average error of biometric measurements between the virtual and real human bodies.

	Height (cm)	Neck to Hip Distance (cm)	Shoulder Width (cm)	Waist Length (cm)	Hip Length (cm)	Arm Length (cm)	Leg Length (cm)
In [8] with two Kinect	1.0	2.4	1.9	6.5	4.0	3.2	2.2
In [5] with two Kinect		2.5	1.5	6.2	3.8	3.0	2.1
In [6] with one Kinect		2.1	1.0	3.2	2.6	2.3	3.1
In [9] with one Kinect		3.7			3.3	1.4	
Ours with one Kinect	0.5	2.0	1.2	3.1	2.2	1.4	1.4

**Table 2:** Average running time spent during each step in the thorough scanning and modeling procedure.

In [6] with One Kinect (CPU Intel Xeon 2.67 GHz, 12 GB RAM)		In [7] with One Kinect (CPU Intel i7-930 2.8 Ghz, 4 Cores, 12 GB RAM)		Ours (CPU Intel i5-2450 m 2.5 GHz, 4 GB RAM)		
Steps	Time (s)	Steps	Time (s)	Steps	Time (s)	Time Complexity
Super-resolution	28	Scanning with ICP registration	113	Data capture	99	
Rigid	110	Poisson fusions	130	Segmentation and denoising	15	O(n)
Non-Rigid	620	Background segmentation	22	Local rigid alignment	85	O(tnlogn)
Poisson	68	Rigid alignment	23	Pre-alignment of multi-view	3	O(nlogn)
		Nonrigid alignment	126	Rigid alignment of multi-view	62	O(tnlogn)
		Albedo extraction	120 (in Matlab)	Non-rigid alignment of multi-view	200	$O\left(tn(\log n+m^{\frac{2}{3}})\right)$
		Visual hull	14	Watertight reconstruction	21	O(n)
		Final watertight fusion	119	Texture mapping	100	O(plogn)
		Poisson texture blending	180			
Total	826	Total	847	Total	585	
